# Spatial shifts in swiping actions, the impact of “left” and “right” verbalizations

**DOI:** 10.1007/s00221-022-06348-0

**Published:** 2022-03-29

**Authors:** Raimey Olthuis, John van der Kamp, Koen Lemmink, Simone Caljouw

**Affiliations:** 1 Center for Human Movement Sciences, University Medical Center Groningen, University of Groningen, Groningen, The Netherlands; 2 Department of Human Movement Sciences, Faculty of Behavioral and Movement Sciences, Vrije Universiteit Amsterdam, Amsterdam, The Netherlands

**Keywords:** Verbalization, Semantics, Aiming, Swiping, Conscious control, Target Visibility, Planning–control

## Abstract

Movements are often modulated by the meaning of cue words. We explore the interaction between verbal and visual constraints during a movement by investigating if spoken words during movement execution bias late movement control of swiping actions on a tablet when vision of the target is removed during the movement. Verbalization trials required participants to vocalize the spatial directions ‘LEFT’, ‘MIDDLE’, or ‘RIGHT’ of the active target, relative to the other presented targets. A late influence of semantics emerged on movement execution in verbalized trials with action endpoints landing more in the direction of the spoken word than without verbalization. The emergence of the semantic effect as the movement progresses reflects the temporal unfolding of the visual and verbal constraints during the swiping action. Comparing our current results with a similar task using a variant verbalization, we also conclude that, larger semantic content effects are found with spatial direction than numerical magnitude verbalization.

**Supplementary Information:**

The online version contains supplementary material available at 10.1007/s00221-022-06348-0.

## Introduction

Although it is not common to see people engage in out loud self-talk in day-to-day life, athletes can often be seen on the court or in the field having, sometimes quite animated, conversations with themselves. Self-talk in the form of intentional cue words is used as a cognitive monitoring strategy aimed at facilitating learning and enhancing sport task performance. Otherwise put, self-talk can act as a sort of guide (Hatzigeorgiadis et al. 2011; Theodorakis et al. 2000). For example, in a dart throwing task, participants that overtly called out ‘CENTER-AIM’ were more successful in aiming to the center of the dart board than participants that performed the same action without self-talk (Jabbari et al. 2019). This supports that self-talk, typically involving spatial directions or content, influences the detection and use of information for guiding movements, and subsequently performance. Along these lines, in a seminal study, Gentilucci and Gangitano (1998) found that the kinematics of reaching and grasping movements toward rods labeled ‘LONG’ or ‘SHORT’, were biased according to the semantic content of the label. Similarly, semantic content has also been seen to directly impact ensuing movements when vocalizing target-related judgments during action. Olthuis et al. (2017) found that when participants called out ‘FURTHER’ while hitting a ball to a target, impact velocity was larger, leading to a farther landing point, than when they called out ‘CLOSER’. Thus, verbally expressing words related to spatial target properties during action was shown to result in movements increasingly prone to the meaning of the vocalized content. It is clear that verbalizations can influence kinematics, however the pre-conditions required for this to happen are largely debated. While much of the findings on the influence of verbalization on movement execution has been interpreted as support for the planning–control model (e.g., Gentilucci & Gangitano 1998; Glover 2004; Glover & Dixon 2002b; Glover et al. 2004; Lindemann et al. 2006), it is also possible that these results can be explained by the constraint-led perspective, that verbalizations impact actions depending on the interaction of task-related constraints, in particular visual information (Newell 1986).

The body of evidence supporting the role of verbalizations in action being explained through the planning–control model has primarily relied on empirical observations from studies where participants are able to use vision to detect and use spatial information about the target throughout the whole action. According to the planning–control model, two different internal processes, an early planning process and a late control process, are said to be involved in executing movements (e.g., Glover 2002, 2004; Glover & Dixon 2001, 2002a). This perspective claims that the early planning process, which occurs prior to movement onset and where a motor program is selected, depends on ventral or allocentric representations that are tightly integrated with cognitive processes (i.e., memory, semantics), while the control process, which predominates during movement execution, relies more on dorsal or egocentric representations that are relatively isolated from cognitive processes. This model posits that cognitive processes, such as semantics, interact with visual information to determine planning but not control. Any biases arising from semantic content in verbalizations generated during planning, can therefore be corrected online as the movement unfolds by the visually based control processes. Claiming support for the planning–control model, Glover and Dixon (2002b) instructed participants to reach and grasp toward target blocks embossed with the word ‘LARGE’ or ‘SMALL’. Semantic content effects were evident in the early stages of the action, which dissipated as the hand approached the continuously visible object. Similarly, when presented with words (e.g., ‘GRAPE’ vs ‘APPLE’) prior to action, larger grips were recorded in the initial reach phases toward targets following presentation of the larger object word ‘APPLE’ compared to ‘GRAPE’. Ultimately, however, target-appropriate grip width was achieved irrespective of the presented word, presumably reflecting adjustments in grip aperture based on the online control process (Glover et al. 2004). In short, this line of research supports that the semantic content of verbalizations/words primarily impacts the early planning stage of action. The ensuing control process, however, is relatively immune to effects of semantic content, since it primarily relies on visual information. A corollary is that once the movement has started, any influence of verbalization largely originates from prior movement planning, unless the movement is re-planned.

An alternative theoretical framework for explaining the effects of verbalization is the constraint-led perspective (e.g., Newell 1986; Davids et al. 2008). Here it is argued that the unfolding movement emerges from self-organizing interacting individual, environmental, and task constraints (Newell 1986). None of these constraints has logical priority, and hence, movement kinematics or patterns are not attributed to individual, internal processes, such as planning and control. The interaction of constraints is dynamic in nature, constantly changing (Gagen and Getchell 2004, 2006; Newell and Jordan 2007), to which performers adapt to find functional movement solutions (Chow et al. 2006). In this framework, semantic content from instructions or verbalizations is considered a key task constraint (Newell and Ranganathan 2010). Yet, it impacts the unfolding movement not by an (a priori) prescription, but in the immediate dynamic interaction with other constraints, such as vision (Handford et al. 1997). The observed dissipation of semantic effects in grip aperture would then reflect the changing interaction between verbal and visual constraints during the unfolding grasping action. Unlike the planning–control model, where verbalization influences movement indirectly through the planning process, the interaction of constraints explanation also allows for semantic effects occurring late in the action, especially when visual information becomes unavailable during action.

When verbal constraints are in the form of specific iterated words, the emergence of task-relevant solutions may include biases specific to the semantic content of the vocalized words. This has been demonstrated in studies where verbalization or presentation with spatial or action-related words has impacted concomitant movements, while influences were not evident when the movements were performed with non-spatial or action-unrelated words (Fargier et al. 2012; Gentilucci et al. 2000; Olthuis et al. 2017). Thus, for verbalization to wield influence on the unfolding movement, the performer must vocalize semantic content that seemingly is connected directly to the movement. Importantly, the impact of verbal constraints may endure or even increase after movement onset, if other task constraints are manipulated; for instance, in the case that visibility gets obstructed. Supporting this assertion, effects of semantics at movement endpoint have been evidenced in studies performed with limited target visibility during action (Olthuis et al. 2017; Rossetti et al. 1995; Rossetti & Régnier 1995, see also Rossetti 1998). In this study, we further explore the interaction between verbal and visual constraints by examining if the introduction of verbalization with spatial directional content during movement execution results in an enlarged directional bias at movement endpoint when also vision of the target is removed during performance of a swiping action. We do this by following up an earlier study.

In that previous study (Olthuis et al. 2021), we used a swiping action as per the current study. Participants were required to verbalize a number that was assigned based on the ordinal location of the target (from one to seven) amidst six other potential targets located in sequence along a semi-circle. In this earlier study, targets were visible for approximately a quarter of the movement duration, and target numbers were called out during movement execution. Given this research included both the removal of vision and introduction of the verbalization during the action, we expected semantic biases to be evident at movement endpoint. However, verbalization was not found to affect movement endpoints under these particular task constraints. We hypothesize that the absence of semantic effects may be related to the specific content of the verbalizations. Possibly, the semantic impact of calling out a number arbitrarily related to target location was not sufficiently connected to the movement to cause interference.

Here, we re-address this issue by keeping all relevant task constraints identical to our previous study (Olthuis et al. 2021), with the exception of the semantic content within the verbalization condition. Similar to Olthuis et al. (2021), for each trial, three consecutive targets, including the active target, will be more salient than the remaining four targets. However, in the present study, instead of calling out target order number in the semi-circle, participants will vocalize the spatial directions ‘LEFT’, ‘MIDDLE’, or ‘RIGHT’ depending on the active target location relative to the other presented targets. This verbalization is more spatially connected to the action as it is semantically related to both the relative direction of the unfolding movement and the location of the target in relation to the other targets at movement endpoint. With our targets presented in a semi-circle array, the verbalizations are expected to result in a semantic content effect, that is, a movement bias in the direction of the verbalized word. This will be observed in an increase in direction error (i.e., movement endpoints to the left and right of the target, for ‘LEFT’ and ‘RIGHT’ verbalizations, respectively). If, in accordance with the planning–control theory, semantic content influences movement indirectly via a priori movement planning processes, with minimal to no effects on subsequent movement control processes, then semantic influences should remain the same or even become absent as the movement progresses. Commensurate with the proposal of a dynamic interaction of constraints, however, it is anticipated that the influence of the verbalization increases in the absence of vision, with the semantic effect strengthening as the movement progresses. To this end, we determine how the semantic effect, if any, unfolds as the movement progresses by measuring the impact of verbalization at 25%, 50%, 75% and 100% of the movement extent. Since, similar to Rossetti and colleagues (Rossetti et al. 1995; Rossetti and Régnier 1995, see also Rossetti 1998), visibility of the targets is removed and verbalization is introduced during the swiping movement, we expect the semantic effect of the verbalization to become evident toward the later segments of the movement extent.

## Methods

### Participants

Twenty right-handed, young adults (16 females and 4 males) aged 20.9 ± 1.5 years participated in the study, 10 per group. A power analysis using G*Power 3.1.9.7 (Faul et al. 2007) indicated that a sample of 14 participants would be required to provide a moderate effect size (*f *= 0.25) with alpha value of 0.05, and power of 0.95. All participants were in good health and functionally able to complete the task without fatigue. They had no known history of visual or neuromuscular deficits and normal or corrected-to-normal vision. Participants did not receive financial compensation for participating in the experiment. Approval from the local ethics committee was granted and a written informed consent from each participant was acquired after explanation of the task and experimental procedures.

### Apparatus and task

Apparatus, procedure, size and location of the targets were the same as in Olthuis et al., (2021) and explained in further detail in that study. A 12.9-inch iPad Pro (Apple Inc., Cupertino, CA, USA) and Apple Pencil were used for this study. The full movements of the Apple Pencil on the iPad Pro were recorded and sent to the iPad Pro with a tracking frequency of 240 Hz in combination with the ProMotion technology of the iPad Pro.

**Fig. 1:**
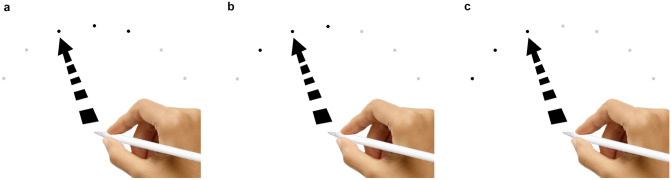
Aiming to Target-C, position-left (**a**); Target-C, position-middle (**b**); Target-C, position-right (**c**). In this figure the target that is indicated by the arrow is the active target, which appeared as red target in the actual trials, to distinguish itself from the other targets

Once the Apple Pencil made contact with the home position a semi-circular global array of seven targets appeared. Each target was equidistance from the home position. The targets in the global array will hereafter be referred to as Target-A—Target-G. All targets and the home position had a diameter of 16 px (see array example, Fig. 1).

The actual or active target within a trial was indicated by color (solid red), positioned either beside or flanked by, two dark gray targets (i.e., gray value of 40%), forming a local array of three targets. In other words, within the local array of three targets, the active target could be positioned to the left of, in the middle of, or to the right of the other two targets; this position of the active target differed across trials. The four remaining targets in the global array of seven targets were light gray (i.e., gray value of 4%) and only moderately visible (Fig. 1—the target that is indicated by the arrow in this figure is the active target, which appeared as red in the trials). Only movements toward the three middle targets of the global array, Target-C, Target-D and Target-E, were included in the analysis because these are the only targets that could occupy all three positions within a local array (i.e., left, middle, and right).

Participants were given 2 s of visibility to locate the active target after which an audio stimulus signaled the participants to begin the movement. To ensure that visual information could not be used for correction of endpoint errors, all targets were visually removed after the stylus left contact with the home area that was defined as a 50-pixel radius around the home target. On average, participants left the home area within 23.25% (± 2.66%) of the total movement time. Thus, vision of the targets was available for approximately the first 23% of the total movement time. Trials were marked as incomplete if the stylus left the home target prior to the auditory start stimulus, or if the endpoint was > 90% of the inter-target distance away from the active target. In trials with the aforementioned violations, an error auditory signal sounded and the trial was aborted, these violations were not included in the analysis.

### Procedure and design

Participants were instructed to place the stylus in the home position when they were ready to start and to keep the stylus steadily placed there until the auditory go stimulus sounded. Once they heard the tone, they were to move as quickly and accurately as possible to the active target location in a single, uncorrected swiping movement, while maintaining contact with the screen. Participants were also told to stop and lift the stylus vertically from the screen when they reached the target, rather than swiping through the target. If participants were unable to spot the active target, they were instructed to move and lift the stylus off the screen next to the home position, so the trial would be recorded as a technical error.

The task fitted in a single session of approximately one hour. All performers completed the task under two different conditions: a no-verbalization (control) condition and a verbalization condition. The verbalization content in this study is the only alteration from Olthuis et al. (2021). In the current study, the verbalization condition required participants to call out in Dutch the location of the active target, relative to the other targets in the local array, ‘LINKS’ (Left) for position-left, ‘MIDDEN’ (Middle) for position-middle, and ‘RECHTS’ (Right) for position-right, while swiping toward the respective targets, immediately after the auditory tone. A researcher remained in the room for all trials to ensure the task was being performed as explained and to verify that the verbalization occurred during the movement.

The session began with instructions on the general task requirements, during which the participants performed 24 practice trials. To rule out any effects of initial learning, this was followed by an additional 72 trials of no-verbalization practice. Participants were then divided into two counter-balanced groups, one group starting with the no-verbalization condition and the other group starting with the verbalization condition. Each condition (i.e., no-verbalization and verbalization) consisted of five blocks of 72 trials, resulting in a total of 720 experimental trials. Between each block, participants rested for 1.5 min. After the fifth block of the first condition, participants received a 5-min break before commencing the first block of the second condition. Swiping movements toward Target-C, Target-D and Target-E, which could occupy all three positions within a local array (i.e., left, middle, and right), were analyzed. In each block, Target-C, Target-D and Target-E were each the active target 18 times, thus up to 540 trials were included in the analysis per participant. The order the targets, their local arrays presented within each block were random.

### Data collection and analysis

The iPad Pro registered the x and y coordinates in pixels (px) on initial connection with the screen at the home position until immediately before the stylus lost connection with the screen at the movement endpoint. Movement onset was defined as the moment the movement reached 5% of the peak velocity and the endpoint was defined when the movement velocity declined to 5% of peak velocity. In rare cases, the movement ended at a velocity larger than 5% of peak, the end of movement was recorded as the last point before lifting the stylus off the screen. The end positions were analyzed to assess the directional error from the center of a given target. Target direction was defined as the orientation of a straight line from the home position to the target, while movement direction was the orientation of a straight line from the home position to the movement endpoint. Directional error was the angle (in radians) between the target direction and the movement direction. A positive angle indicates a clockwise bias (to the right) and a negative angle indicates a counter-clockwise error (to the left). The directional error in radians was also determined at 25%, 50% and 75% of the movement extent to uncover changes in semantic bias throughout the trajectory.

Before statistically analyzing the dependent variables, technical errors and outliers were excluded. Technical errors were defined as trials where the iPad failed to save the endpoint, or where the distance of the registered endpoint was larger than 90% of the inter-target distance. This may have occurred if the stylus lost contact with the touchscreen during the movement, if the movement was initiated before the go-signal, or if a trial was aborted or aimed toward the wrong target. Values less than Q1-3(IQR) or greater than Q3 + 3(IQR) were considered outliers and removed from analysis. Overall, 1.71% of all trials were eliminated after the technical error analyses and 0.31% of trials were eliminated after outlier analysis. There were no significant differences in the number of technical errors, or the number of outliers, between the no-verbalization and verbalization conditions.

We performed a 3 (global target: C, D, E) by 2 (condition: no-verbalization, verbalization) by 3 (local position: left, middle, right) repeated measures ANOVA. All tests were subjected to Mauchly’s test for sphericity. Whenever the Mauchly’s sphericity assumption was violated, the ANOVA results were adjusted using the Huynh–Feldt adjustment for non-sphericity. For post hoc tests on interactions with targets, we performed repeated measures ANOVAs. Paired *t* tests, with a Bonferroni adjustment of the αlevel, were used for all other post hoc comparisons. Partial eta-squared (*η*_p_^2^) was used to determine effect size for the ANOVAs. Effect sizes were calculated with partial eta-squared (*η*_p_^2^), with values larger than 0.01, 0.06, and 0.14 indicating small, moderate, and large effect sizes respectively (Cohen 1988).

## Results

### Directional error

*Directional error at 100% of movement extent* The ANOVA for constant median directional error (in radians) at movement endpoint revealed main effects for Target [F(2,38) = 26.65, *p < *0.001, *η*_p_^2^ = 0.58] and Position [F(2,38) = 44.53, *p < *0.001, *η*_p_^2^ = 0.70]. A main effect for Condition was not found [F(1,19) = 0.34, *p = *0.57, *η*_p_^2^ = 0.02], but an interaction effect was observed for Condition × Position [F(2,38) = 5.64, *p < *0.01, *η*_p_^2^ = 0.23]. Also, an interaction for Target × Position was found [F(4,76) = 9.52, *p < *0.001, *η*_p_^2^ = 0.33]. No other interaction effects were found (Fs < 1.37 & ps > 0.25).

**Fig. 2:**
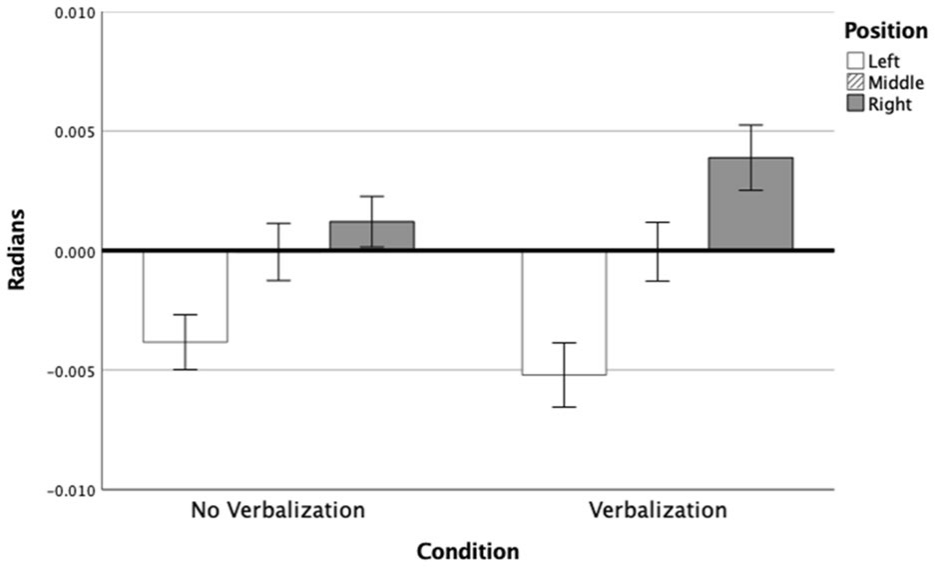
Mean direction error and standard error per Condition per Position at 100% of movement extent

Figure 2 shows the local Position effect for both conditions. Position-left results in a counter-clockwise bias (i.e., to the left) compared to position-right. The post hoc for the Condition × Position effect indicated that calling out “RIGHT” while moving toward position-right increased the clockwise error compared with actions to the same position without verbalization. The same trend can be seen for position-left increasing the counter-clockwise error, although it did not reach significance (*p = *0.25). This indicates that verbalization of a spatial preposition during movement execution can affect motor performance, particularly in the direction of the iterated word (see also Fig. 5D).

**Fig. 3:**
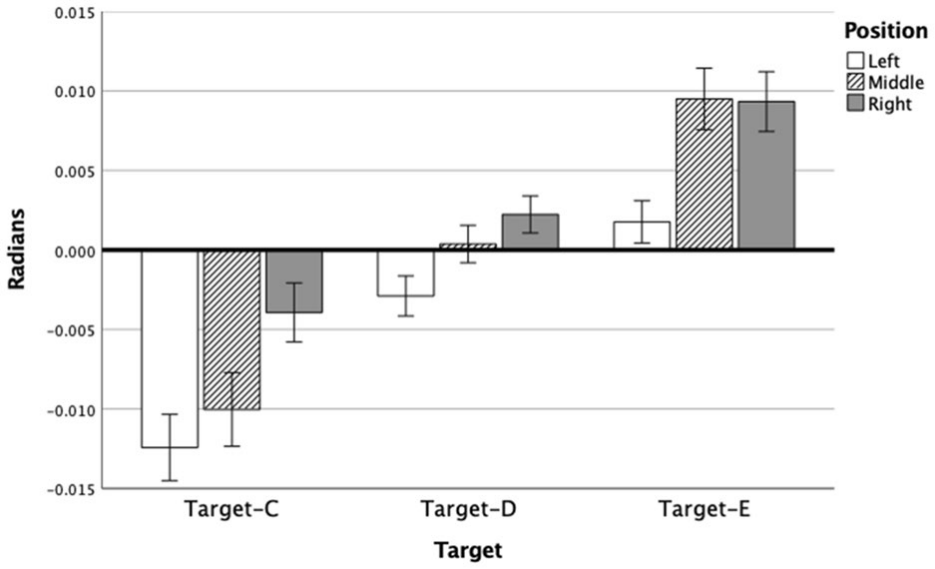
Mean direction error and standard error per Target per Position at 100% of movement extent

Figure 3 presents the global target effect for endpoint bias, post hoc tests indicated that each target is significantly different from one another. Specifically, post hoc comparisons indicated that Target-C holds the most counter-clockwise error, Target-E the most clockwise error and Target-D the least error overall. The local array displays a similar effect for Position, position-left holds the most counter-clockwise error, position-right the most clockwise error and position-middle the least error. Post hoc paired t tests indicated that the Target x Position interaction is associated with Target-E. For this target, position-middle and position-right are not significantly different from one another, while for the other two targets, these positions vary significantly from one another.

*Directional error at 25%, 50% and 75% of movement extent* At 25% of the movement extent, there were no significant main effects of Position [F(2,38) = 2.58, *p = *0.09, *η*_p_^2^ = 0.12], Condition [F(1,19) = 0.51, *p = *0.48, *η*_p_^2^ = 0.03], or Target [F(2,38) = 1.03, *p = *0.37, *η*_p_^2^ = 0.05]. Interaction effects were also not found at 25% of movement extent, Position × Condition [F(2,38) = 0.69, *p* > 0.50, *η*_p_^2^ = 0.04] other interactions (Fs < 2.00; ps > 0.10) (see Fig. 5A).

**Fig. 4:**
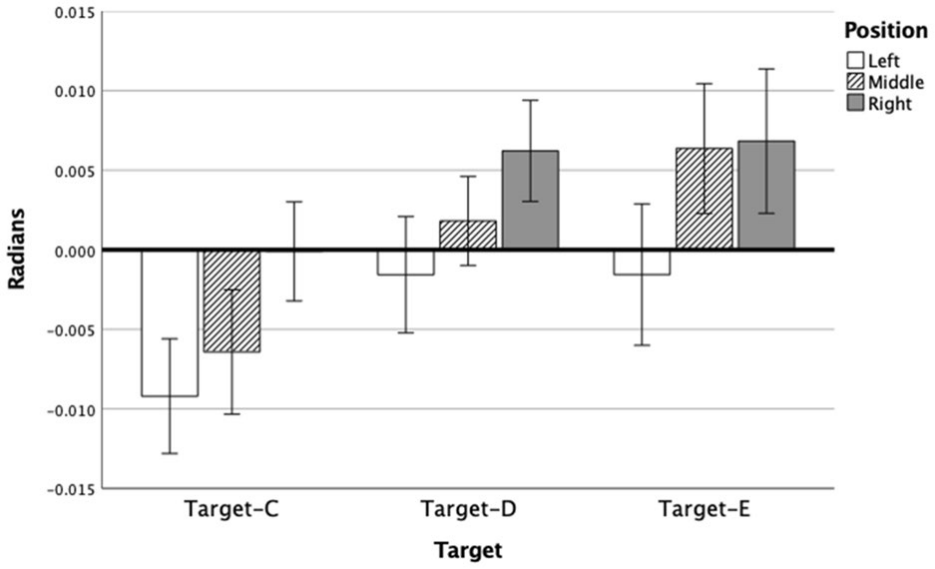
Direction error (Radians) and standard error per Target per Position at 75% of movement

**Fig. 5:**
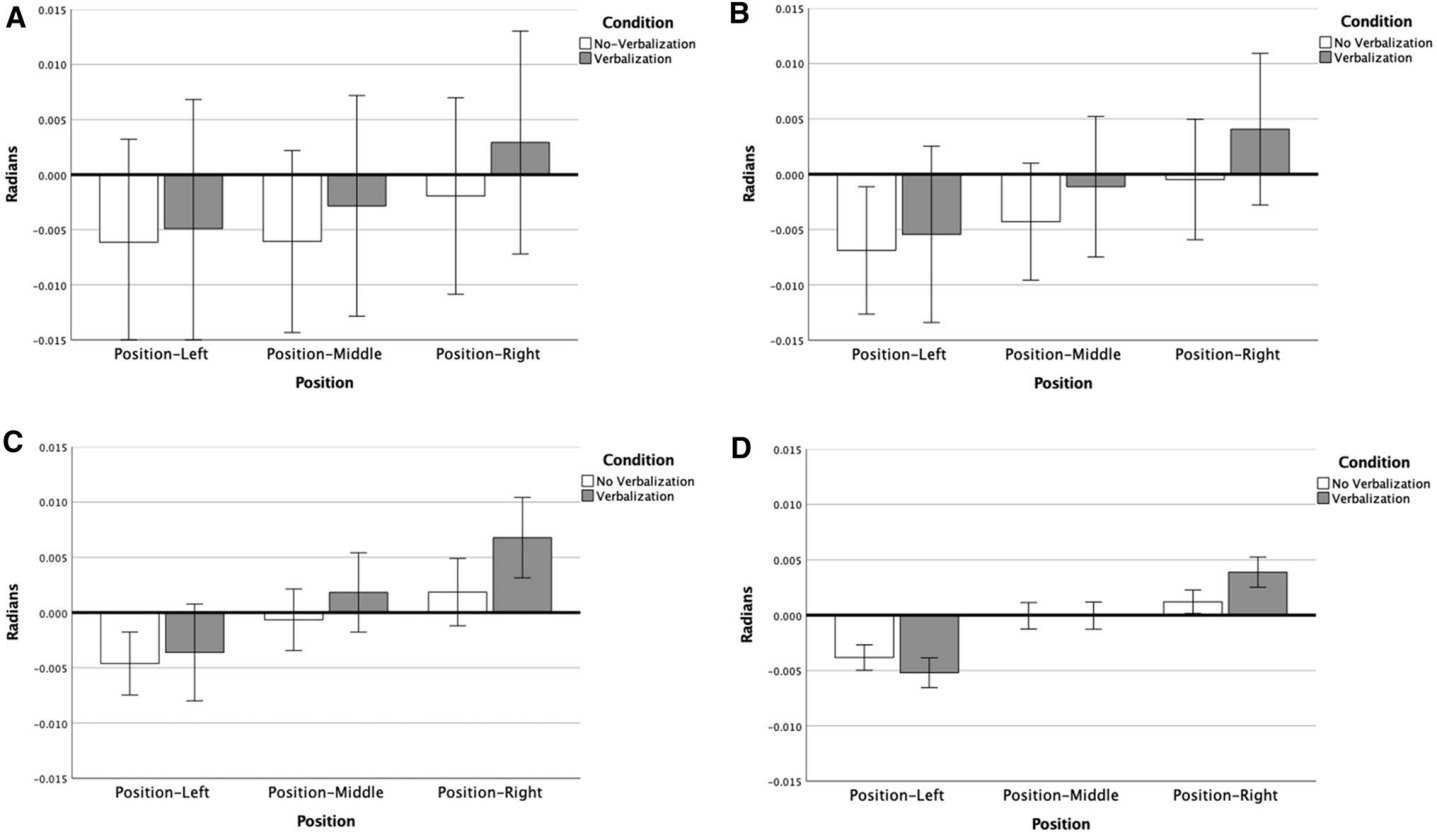
Direction error (Radians) and standard error per Position per Condition at 25% of movement (**A**), 50% of movement (**B**), 75% of movement (**C**), and 100% of movement (**D**)

By 50% of the movement extent, a main effect of Position [F(2,38) = 8.85, *p = *0.001, *η*_p_^2^ = 0.32] had developed, see Fig. 4. However, there were no main effects of Target [F(2,38) = 1.18, *p = *0.32, *η*_p_^2^ = 0.06] or Condition [F(1,19) = 1.59, *p = *0.22, *η*_p_^2^ = 0.08]. There also was not a Position × Condition effect [F(2,38) = 1.08, *p = *0.35, *η*_p_^2^ = 0.05] (see Fig. 5B), or any other Interaction effects (Fs < 2.00; ps > 0.10). In line with the positional bias at the endpoint, post hocs indicated that position-right resulted in significantly more clockwise error than position-middle and position-left.

**Fig. 6:**
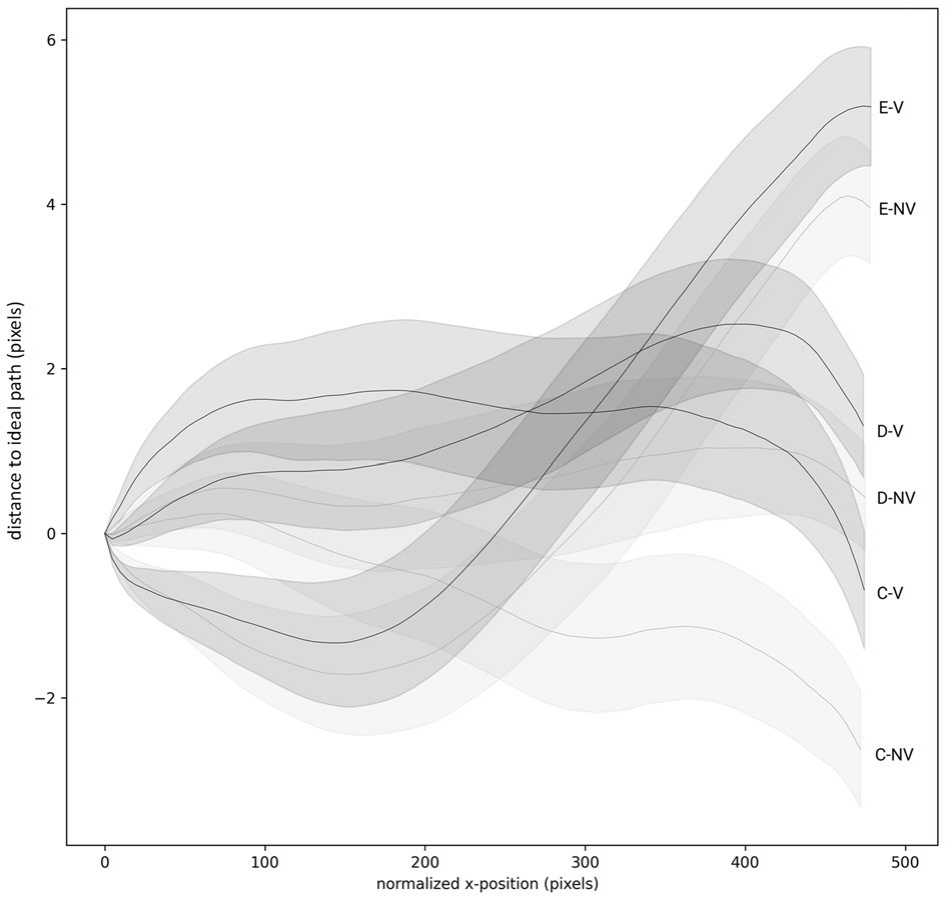
Normalized mean trajectories with 95% confidence intervals, for all participants for both conditions for Position-Right to all three Targets, (*C-NV* Target-C, No-Verbalization, *C-V* Target-C, Verbalization, *D-NV* Target-D, No-Verbalization, *D-V* Target-D, Verbalization, *E-NV* Target-E No-Verbalization, *E-V* Target-E Verbalization)

At 75% of the completed movement, there were main effects for Target [F(2,38) = 6.01, *p < *0.05, η_p_^2^ = 0.24] and Position [F(2,38) = 29.76, *p < *0.001, *η*_p_^2^ = 0.61]. There was also an interaction effect of Target × Position [F(4,76) = 2.66, *p < *0.05, *η*_p_^2^ = 0.12]. Condition remained insignificant [F(1,19) = 3.21, *p = *0.09, *η*_p_^2^ = 0.14] and the interaction of Position x Condition also just failed to reach significance, with a moderate effect size [F(2,38) = 3.05, *p = *0.06, *η*_p_^2^ = 0.14]. No other Interaction effects were found (Fs < 3.25; ps > 0.47). Post hocs indicated that, in line with the effect for the endpoints (i.e., 100%), for all targets, position-left displayed more counter-clockwise error than position-right. Meanwhile, the middle position adapted depending on the target location, counter-clockwise on the left and clockwise on the right (see Fig. 4). The non-significant Position × Condition interaction shows a trend toward the significant interaction found at movement endpoint, with verbalizing ‘RIGHT’ leading to an increased clockwise error than the no-verbalization trial (see Fig. 5C). Thus, the effect reached significance somewhere between 75% and 100% of movement extent. The normalized mean trajectories for position-right show that differences between the no-verbalization and verbalization conditions increase in the later stages of the movement only (see Fig. 6). The effect of movement duration was removed by resampling the path length into 100 evenly divided steps.

Finally, we also assessed verbalization for accuracy and found that there were no errors. For all verbalized trials, participants called out the correct location of the target within the local array.

## Discussion

Verbalization is often a critical constraint on how we perceive and interact with the world. This ability of words to modify and/or influence existing thought and behavioral patterns makes it a powerful cognitive strategy to monitor and direct behaviors and motor performance (Wang et al. 2003; Zinsser et al. 2015). There is increasing evidence that movements may be influenced depending on the semantic content of verbalizations made during action. However, despite increasing research on the associations between verbalization and action, there is still mixed evidence regarding the specific conditions under which these effects are observed, and the limits of these interactions. In a previous study (Olthuis et al. 2021), we observed that verbalizing a number assigned to the absolute target location (‘ONE’ to ‘SEVEN’) during action did not systematically affect movement endpoints. Given the numerical magnitude bias (Bächtold et al. 1998; Fischer 2001), verbalization was expected to result in counter-clockwise endpoint biases for smaller numbers associated with the left targets and clockwise endpoint biases for larger numbers associated with the targets on the right side. However, ultimately no effect was found in conjunction with verbalization. In the current study, we asked if a verbalization effect would be revealed if all relevant task constraints were identical to the previous study, except for replacing the numerical label within the global array verbalization with a spatial label of the active target relative to the other presented targets in the local array (‘LEFT’, ‘MIDDLE’, or ‘RIGHT’). The verbalizations ‘LEFT’ and ‘RIGHT’ are spatially related to both the relative direction of the advancing movement and the endpoint location of the target in relation to the other targets; thus, we expected the verbalization to impel movements counter-clockwise and clockwise of the targets, respectively. Ultimately, we indeed observed a semantic effect at movement endpoint, when participants called out ‘RIGHT’; while moving toward the most rightward position an increased clockwise endpoint error was found compared with actions to the same position without verbalization. A similar but non-significant trend appeared for ‘LEFT’ vocalizations. These semantic effects were exclusively related to position in the local array, without influencing errors to absolute target locations in the global array. Given that the response modality and task set-up were identical in both studies, the revealed effect was the result of replacing the numerical label verbalization with a verbalization associated with a spatial direction within the local array. Thus, the catalyst for the verbal effect on movement execution was related to the particular spatial word being iterated. This supports the need for a close link between the content of the verbalization and its relevance to the action for an effect to occur.

While impact of action–word compatibility on accompanying movements has been demonstrated in the literature (Gentilucci and Gangitano 1998; Gentilucci et al. 2000; Glover and Dixon 2002b; Olthuis et al. 2017; Rossetti et al. 1995; Rossetti and Régnier 1995), it is less clear if these are best explained by the planning–control model or an alternative hypothesis, such as interacting task constraints. Most verbalization studies found related biases only on the initial part of the movement, alluding authors to rely on the planning–control model for explanation (e.g., Gentilucci and Gangitano 1998; Glover and Dixon 2002b; Glover et al. 2004; Lindemann et al. 2006). The planning–control model distinguishes two processes associated with two phases of movement control, an initial planning process, mostly before movement initiation which is susceptible to bias by factors, such as verbalization and, a later, on-line control module which can correct for these initial errors (e.g., Glover 2002, 2004; Glover and Dixon 2001, 2002a, 2002b). Based on this perspective, if the control process is disrupted, by taking away vision, then movements will be executed entirely “as planned” (i.e., without the benefit of on-line control) and the original bias may remain, but would not increase. An increased verbalization effect would theoretically only be possible if the movement was re-planned. In our study, however, for each position, we could not distinguish between verbalization and no-verbalization trials at the beginning of the action. Instead, effects of verbalization became significant as the movement unfolded, which was also attested for by increasing effect sizes. Verbalization trials were seen ending farther from the target in the direction of the spoken word within the last 25% of the movement. Our findings suggest that the effects of verbalizations do not in all circumstances decay as the movement progresses.

Critics supporting the planning–control model may argue that the removal of targets in our set-up could have led to actions being executed entirely “as planned” (i.e., without the benefit of on-line control) and thus cognitive processes, such as semantics, that would not have affected actions in real time may now have influence over these “planned” actions. However, in our set-up, visual information regarding the target was available at movement initiation and until approximately 25% of the movement extent, in addition to information regarding the effector (i.e., hand) being available throughout the entire action. Given this initial visibility of target and insistent visibility of hand, this action is expected to have been executed online and not as planned (Glover 2004; Westwood and Goodale 2003; Tremblay et al. 2013). Further, online control is thought to decay gradually, with little reduction during the first two seconds (Westwood et al. 2001). Targets in our study were only absent on average for 0.41 s, thus we would expect little to no decay of online control. Consequently, according to the planning–control model, any semantic biases arising from verbalizations should have been corrected online as the movement unfolded, yet we identified semantic related biases at movement endpoint. This explanation could potentially lead to another argument, that such a quick action is ballistic in nature and therefore a control phase would not be expected. Since in this study, action corrections are visible in the trajectories as the movement progresses, we argue that this movement is fast, but not ballistic in nature. It may also be suggested, in favor of the planning–control model, that these adjustments are the result of fast re-planning of the movement during execution. On the basis of the current data, we cannot exclude the possibility that verbalization resulted in the inhibition of the previous action and implementation of a new action. However, we contend re-planning would be unlikely since actions were directed at stable targets that were fully visible during the first part of the movement. Re-planning would also be a relatively slow process and unlikely in case of such a fast movement and if present would likely present in a clear disruption/slowing down of the ongoing movement, which was not observed.

Given the increase in verbalization effects observed in the current study, the possibility that kinematic changes emerge from the dynamic interaction of constraints, arguably, appears more convincing than presuming that re-planning took place. This theory posits that movement emerges from self-organizing dynamic or changing interactions between constraints, where all constraints, such as verbalizations, can impact movement at any moment, depending on the interaction of constraints at that time. In this respect, visibility of the target and the timing when verbalization is introduced both warrant further attention. Studies supporting the planning–control method presented verbalization prior to movement initiation and provided full visibility of the targets throughout the entire action (Gentilucci and Gangitano 1998; Glover and Dixon 2002b; Glover et al. 2004; Lindemann et al. 2006). In the present study, and others demonstrating effects at movement endpoint (Rosetti 1998), words were primed/spoken during the trajectory, when the action was taking place and not before and visibility of the targets was not available throughout the full action. When available, vision of a target often guides actions throughout the trajectory, however, when key visual reference points are unavailable actors may vary their behavior to accommodate by adapting to the available information source, here the semantic content of the verbalization. Thus, the ultimate effects of verbalization on the action may depend on the temporally changing interaction of task constraints (i.e., visual and verbal), rather than the phase of movement (i.e., planning versus control). Overall, a self-organizing interacting constraints perspective appears to be the more parsimonious explanation for these effects, rather than presuming semantic effects are mediated by different planning and/or control processes.

While we have found an impact of semantic content on movement kinematics, why this effect occurred in the current and not in our earlier study (Olthuis et al. 2021) remains to be clarified. Two possible, and not necessarily mutually exclusive, explanations can be offered. First, the verbalizations in these studies placed unequal conscious demands on the respective participants. In the earlier study, the numerical assignment was constant for every absolute global target location, consequently for each target, participants always made the same verbalization. However, the spatial direction verbalizations made in the current study were related to the local array and independent of the global target number, so for each target participants had to select and call out one of three possible verbalizations. Thus, in the current study, participants had increasing conscious monitoring requirements compared to the previous study. This increased conscious monitoring could feasibly have led to a higher reliance on allocentric information, leading to an action more susceptible to contextual influence (Goodale and Milner 1992; Hu and Goodale 2000; Olthuis et al. 2017; Westwood and Goodale 2003; Westwood et al. 2000). An alternative explanation is that spatial verbalizations have the capability of influencing both the relative direction of the advancing movement (movement trajectory control) and the location of the target in relation to the other targets at movement endpoint (movement endpoint control), whereas numerical label verbalizations are exclusively related to the movement endpoint (movement endpoint control). Thereby, the semantic effect may be amplified in this study compared to Olthuis et al. (2021) as a result of spatial direction verbalizations being highly relevant to the concomitant action.

Beyond the spatial biases inflicted by verbalization, we also observed systematic spatial biases related to the location of the target within the global and local target arrays. At movement endpoint, there was an effect of Target and Position as well as an interaction effect between them. The main effects of Target and Position were as expected and consistent with earlier findings (Olthuis et al. 2021). The similarity of the findings in the present study with those observed in Olthuis et al. (2021) is noteworthy as this supports our conjecture that the Position effect found with verbalization in the current study relates to the semantic content of the verbalized words.

Overall, we postulated that verbalizing the spatial direction of the target location in relation to its surroundings during movement execution would induce an effect, in particular with movement bias in the direction of the spoken word (i.e., ‘LEFT’ or ‘RIGHT’). Indeed, our results showed an influence of semantics on movement execution, which seems to have gradually emerged throughout the movement. Thus, our results provide evidence that semantic effects can still remain apparent at movement endpoint. This is an intriguing finding that is most coherently explained as arising from the temporarily unfolding dynamic interaction of movement constraints.

## Supplementary Information

Below is the link to the electronic supplementary material.

Supplementary file1 (XLSX 15 KB)

## Data Availability

The datasets generated during and/or analyzed during the current study are available from the corresponding author on reasonable request.
